# A Case of Actinomycosis

**Published:** 1886-12

**Authors:** A. J. Ochsner

**Affiliations:** 300 South Wood street


THE CHICAGO

MEDICAL JOURNAL AND EXAMINER.

VOL. LIII. DECEMBER, 1886. No. 6.

### ORIGINAL COMMUNICATIONS.

### REPORT OF A CASE OF ACTINOMYCOSIS. *By* A. J. OCHSNER, M.D.

[Read before the Chicago Medical Society on November 1st.]

Until the autumn of 1877, this patient, aged 56 years, was in perfect health, following his occupation of stock-raising and dealing in livestock. At this time he was engaged in shipping stock to the east. During a very fatiguing journey and much exposure to draughts of cold air, the patient experienced severe pain in the left antrum of Highmore, which he supposed to be neuralgia caused by defective teeth. He consequently had removed from his upper jaw seven teeth which proved to be sound, and he secured no relief. All the other teeth of his upper jaw had previously been removed at different times, having been more or less decayed.

For about six months the patient suffered excruciating pain in the left antrum and in both eyes, continuing each day from sunrise until sunset.

Early in 1878 there was a spontaneous opening of the abscess into the pharynx, the sinus closing and re-opening repeatedly, each time evacuating a considerable amount of pus and some blood, and giving the patient marked relief.

A portion of the discharge usually entered the larynx at night, giving rise to severe cough.

During the month of May, 1880, the patient underwent a surgical operation, consisting in making an opening through the mouth into the antrum above the first molar, curetting and irrigating the cavity. The irrigation was continued by the patient two or three times daily for two years. During all of this time he suffered severely from pain and weakness.

In the spring of 1882, the patient went to the northern part of Mexico and spent the summer on the plains and among the mountains between this point and Colorado, returning in the autumn to Indiana, with the antrum closed and his general health much improved.

Later during the same autumn he returned to Mexico and remained there in comparatively good health for twenty months. At this time, however, he began to suffer from a sensation of suffocation, which still continues.

In July, 1885, the patient began to cough, the cough subsiding somewhat under treatment, but increasing to an alarming extent in July, 1886, and continuing to the time of admission into the Presbyterian Hospital of this city, October 13th, 1886.

During the months of September, 1885 and 1886, he expectorated blood, but he thinks it came from the posterior nares.

Since the beginning of October, 1886, he has again expectorated mucus and pus, streaked with blood, which undoubtedly comes from the lungs or the bronchi.

He has lost thirty-seven pounds in weight during the past two years. His position is stooping, the chest is full in front and there is a decrease of motion on the left side, with dullness, roughened respiratory sounds and numerous mucous râles. Below the upper border of the fifth rib and throughout the right side the sounds are normal.

The above history led me to suspect actinomycosis of the left lung, having primarily existed in the antrum. On making a microscopic examination of the sputum, the characteristic fungus was at once found, confirming the diagnosis, of course, beyond a doubt.

The following facts may be of practical interest in connection with this case.

He has been engaged in raising, buying and selling, and handling large numbers of cattle for more than forty years. Among these animals there were many suffering from the disease known as lumpy-jaw, and it was the practice of the patient to cure the animals thus affected, by freely opening the abscess by crucial incision, extirpating as much as possible of the lump and introducing about one drachm of powdered arsenic into the cavity. Repeating this once or twice, usually effected a permanent cure.

300 South Wood street.

